# Sepsis biomarkers in unselected patients on admission to intensive or high-dependency care

**DOI:** 10.1186/cc12588

**Published:** 2013-03-26

**Authors:** Martin J Llewelyn, Mario Berger, Mark Gregory, Ravi Ramaiah, Amanda L Taylor, Ingo Curdt, Frédéric Lajaunias, Rolf Graf, Stuart J Blincko, Stephen Drage, Jonathan Cohen

**Affiliations:** 1 Brighton and Sussex Medical School, Falmer, BN1 9PS, UK; 2 Brighton and Sussex University Hospitals NHS Trust, Brighton BN2 5BE, UK; 3 Abbott GmbH & Co.KG, Abbott Diagnostics Division, Discovery Research, Max-Planck Ring 2, 65205 Wiesbaden, Germany; 4 Lascco SA, Frédéric Lajaunias, 8 Rue de la Rôtisserie, 1211 Geneva 3, Switzerland; 5 Klinik für Viszeral- and Transplantationschirurgie, Rämistrasse 100, Universitätsspital Zürich, 8091 Zürich, Switzerland

## Abstract

**Introduction:**

Although many sepsis biomarkers have shown promise in selected patient groups, only C-reactive protein and procalcitonin (PCT) have entered clinical practice. The aim of this study was to evaluate three promising novel sepsis biomarkers in unselected patients at admission to intensive care. We assessed the performance of pancreatic stone protein (PSP), soluble CD25 (sCD25) and heparin binding protein (HBP) in distinguishing patients with sepsis from those with a non-infective systemic inflammatory response and the ability of these markers to indicate severity of illness.

**Methods:**

Plasma levels of the biomarkers, PCT and selected inflammatory cytokines were measured in samples taken from 219 patients during the first six hours of admission to intensive or high dependency care. Patients with a systemic inflammatory response were categorized as having sepsis or a non-infective aetiology, with or without markers of severity, using standard diagnostic criteria.

**Results:**

Both PSP and sCD25 performed well as biomarkers of sepsis irrespective of severity of illness. For both markers the area under the receiver operating curve (AUC) was greater than 0.9; PSP 0.927 (0.887 to 0.968) and sCD25 0.902 (0.854 to 0.949). Procalcitonin and IL6 also performed well as markers of sepsis whilst in this intensive care unit (ICU) population, HBP did not: PCT 0.840 (0.778 to 0.901), IL6 0.805 (0.739 to 0.870) and HBP 0.607 (0.519 to 0.694). Levels of both PSP and PCT reflected severity of illness and both markers performed well in differentiating patients with severe sepsis from severely ill patients with a non-infective systemic inflammatory response: AUCs 0.955 (0.909 to 1) and 0.837 (0.732 to 0.941) respectively. Although levels of sCD25 did not correlate with severity, the addition of sCD25 to either PCT or PSP in a multivariate model improved the diagnostic accuracy of either marker alone.

**Conclusions:**

PSP and sCD25 perform well as sepsis biomarkers in patients with suspected sepsis at the time of admission to intensive or high dependency care. These markers warrant further assessment of their prognostic value. Whereas previously published data indicate HBP has clinical utility in the emergency department, it did not perform well in an intensive-care population.

## Introduction

Sepsis is a common reason for admission to intensive care and high dependency units and the incidence of sepsis continues to rise [1]. The symptoms and signs of sepsis are highly variable and this makes clinical recognition and assessment of severity difficult. At key decision points, such as at the point of admission to high dependency care, doctors are confronted with conflicting pressures. On one hand there is a drive to minimise unnecessary antibiotic prescribing and on the other, there is compelling evidence that timely and specific administration of antibiotics saves lives [2]. Biomarkers can inform decision making in this situation both by indicating the presence or absence of infection in a patient who has a systemic inflammatory response syndrome (SIRS), and as measures of severity or prognosis [3].

A recent review of the sepsis biomarker literature identified 178 biomarkers evaluated in 3,370 studies and yet only two have become widely used [4]. C-reactive protein (CRP) has been in use for over 20 years but has poor specificity unless high cut-off levels are used. Procalcitonin (PCT) appears to be more specific for infection and, unlike CRP, its levels appear to reflect severity and prognosis. However, a recent meta-analysis challenged this [5] and subsequent controlled trials of PCT-guided treatment of sepsis have produced conflicting results [6,7]. There remains an urgent need for better markers of sepsis.

A major challenge in evaluating new sepsis biomarkers is the lack of any gold-standard test. Consequently, early evaluation of biomarkers tends to involve very tightly clinically-defined patient groups which do not reflect the heterogeneity which clinicians encounter. Several novel biomarkers have recently demonstrated promise in the early evaluation of patients for sepsis and the aim of this study was to further evaluate some of the most promising in unselected patients presenting to the ICU. This approach was taken to provide a challenging, real-life assessment of these markers to screen for those which perform well enough to justify further evaluation in clinical trials. We selected three novel candidate markers. Pancreatic Stone Protein (PSP), also known as regenerating protein and lithostathine, is a lectin-binding protein, the blood levels of which increase in inflammation. It is a secretory protein produced by pancreatic acinar cells and intestinal Paneth cells but its function is not clear. Elevated levels occur in acute and chronic pancreatitis, chronic renal failure and gastrointestinal malignancy [8,9], but among trauma patients, levels of PSP rise when sepsis develops [10]. High PSP levels at the onset of ventilator-associated pneumonia and in patients with septic shock predict mortality [11,12]. CD25 is the IL2 receptor alpha chain and is expressed constitutively on regulatory, FOXP3+, T cells. It is also expressed on effector T cells following activation and may reflect the development of a compensatory regulatory response [13]. Soluble CD25 (sCD25) has been found to be higher in patients with sepsis than in patients with non-infective SIRS [14]. Heparin Binding Protein (HBP) is an inflammatory mediator contained within neutrophil secretory and azurophilic granules which demonstrated superior sensitivity and specificity to PCT and CRP in identifying patients with severe sepsis in the emergency department [15]. Recent ICU-based studies suggest HBP levels may commonly be elevated among ICU patients and reflect the presence of cardiovascular shock [16,17]. Data on prediction of mortality are conflicting [16-18]. Here we assessed, first, the performance of these markers in distinguishing patients with sepsis from those with a non-infective systemic inflammatory response and, secondly, the ability of these markers to indicate severity of illness.

## Materials and methods

### Study population

We sought to enrol all admissions to the general intensive care unit (ICU) (17 beds) and high dependency unit (HDU) (8 beds) at Brighton and Sussex Hospitals NHS trust between August 2010 and January 2011. Patients were excluded if they were under 18 years of age or where it was not possible to obtain patient consent or consultee approval to enrol the patient within six hours of admission. The ethical approval for the study defined a consultee in accordance with the United Kingdom Mental Capacity Act as a friend or relative willing to advise on the likely wishes of the patient.

The study was approved by the North Wales Research Ethics Committee (Central and East) reference number 10/WNo03/19. Written informed consent or consultee approval to enrol was secured for all participants in the study. All data were anonymised.

We collected baseline characteristics of the patients including demographic information, Sequential Organ Failure Assessment (SOFA) score in the first 24 hours, comorbidities, site and type of infection, and blood tests. Patients were followed up until discharge from the ICU/HDU or death.

Blood was collected from patients within six hours of their admission to the unit. Samples were taken into sodium citrate tubes, centrifuged and plasma was stored at -80°C until the end of the study when all samples were analysed for each marker as a single batch.

### Definitions

The 2001 International Sepsis Definitions Conference definitions of Systemic Inflammatory Response Syndrome (SIRS) and sepsis were used [19], sepsis being defined as SIRS plus either proven infection (on the basis of microbiological sampling or radiology) or probable infection (considering the patient's clinical presentation, white cell count, CRP, radiology) and non-infective SIRS being defined as SIRS associated with an established underlying non-infective diagnosis and no reason to suspect any on-going infection. Categorisation of subjects was made independently by two members of the study team (ML and SD) blind to the biomarker results and any disagreements were resolved by discussion. A patient's sepsis or non-infective SIRS was defined as being severe if it was associated with organ dysfunction (SOFA score of 2 or more for any organ system) [19,20]

### Cytokine and biomarker measurements

Cytokine levels (IL6, IL8, IL1β, granulocyte macrophage colony stimulating factor (GMCSF), TNFα) were measured on a Luminex LX200 using Invitrogen's Human Inflammatory 5-Plex panel (Invitrogen/Life Technologies, Darmstadt, Germany) and Millipore filter plates (VWR, Darmstadt, Germany) as per the manufacturers' instructions. PSP and HBP were measured on microplate assays as previously described [15,20]. PCT was measured on a Kryptor instrument (Brahms, Henningsdorf, Germany). Levels of sCD25 were measured on commercially available microplate assays (Human IL-2 sRa (sCD25) OptEIA Set, Becton Dickenson, San Diego, CA, USA). All biomarker analyses were conducted blind to the clinical data.

### Statistical analysis

Continuous variables were described using the mean ± SD for normally distributed data or the median (interquartile range (IQR)), for non-normally distributed data. Comparisons of group differences for continuous variables were made by one-way ANOVA or Mann-Whitney test as appropriate. Categorical data were described as the number of patients in each category with corresponding percentages. The significance of differences in proportions was tested by Chi-squared test.

The performance of each marker in identifying sepsis or severe sepsis was assessed as area under a receiver operating characteristic (ROC) curve. For each marker ROC curves were used to derive cut-offs for sensitivity and specificity in distinguishing sepsis from non-infective SIRS.

To establish the potential for combinations of markers to improve identification of sepsis and severe sepsis, we first used univariate correlation (Spearman's rho) to assess the relationship between the markers. Since the majority of continuous variables were non-linear, associations between levels of each parameter and sepsis or severe sepsis were sought after dividing the values for each marker into quartiles. Stepwise logistic regression was then used to assess the impact of different combinations of markers on differentiation of sepsis from non-infective SIRS. Statistical analyses were performed in SPSS 17.0 (IBM Corporation Somers, NY, USA) and Prism 5 (GraphPad Software Inc. La Jolia, CA, USA). All *P-*values were two-sided and statistical significance was set at an α-value of 0.05.

## Results

### Base-line characteristics and outcome of the study population

Between August 2010 and January 2011, 486 patients were admitted to the HDU/ICU. For 267 no study blood sample could be obtained within six hours of admission because consultee approval could not be obtained in time. These patients did not enter the study. Of the 219 patients enrolled, 34 were admitted to the HDU and 185 to the ICU. The median age (IQR) of the patients was 65.9 years (52.0 to 76). Of these, 93 (42%) were female. Twenty patients (9.1%) died on the ICU. A total of 115 subjects (52.5%) were surgical patients. The median length of stay was three (one to six) days.

A total of 198/219 patients (91.8%) met two or more of the SIRS criteria in the first 24 hours of admission to ICU. Interrater reliability for classification of these as sepsis or having a non-infective aetiology was high (Cohen's kappa 0.8) but in 36 patients (18.2%) it was not possible to determine with certainty whether infection was present or not. This group included four patients with pancreatitis. A total of 87/198 patients with SIRS (43.9%) were deemed to have sepsis, while 75 (37.9%) were deemed not to have infection and were thus classified as having non-infective SIRS. Demographic and clinical features of the different patient groups are shown in Table 1. Among patients with a systemic inflammatory response, those with organ dysfunction were less likely to be on the HDU, were less often post-surgical patients and had higher SOFA scores, but overall, patients with sepsis, non-infective SIRS and SIRS, which could not be categorised as sepsis or non-infective, were similar in terms of age, gender and severity of illness.

**Table 1:** Base-line characteristics and outcome of patients included in the study

Variable	SIRS criteria not fulfilled	SIRS not categorised as sepsis or non-infective	Non-infective SIRS		Sepsis	
			
			Without organ dysfunction	With organ dysfunction	Without organ dysfunction	With organ dysfunction
**N**	21	36	25	50	11	76

**Age (years) (IQR)**	72.1 (61.5 to 75.4)	66.8 (40.5 to 77.5)	63.3 (51.2 to 71.5)	62.6 (51.0 to 74.5)	65.1 (42.6 to 73.4)	66.7 (54.3. to 75.8)

**Female, n (%)**	5 (29%)	19 (53%)	11 (44%)	21 (42%)	6 (54%)	30 (39%)

**HDU, n (%)**	6 (33%)	4 (11%)	11 (44%)	4 (8%)	4 (36%)	4 (5%)

**Surgical**	14 (78%)	(36%)	20 (80%)	19 (38%)	10 (91%)	36 (47%)

**SOFA score Day 1 (IQR)**	4 (3 to 7)	5 (3 to 8)	1 (1 to 2)	6 (3 to 8)	2 (1 to 3)	7 (5 to 10)

**Length of stay on ICU/HDU (days) (IQR)**	2.8 (1.3 to 8.3)	3.4 (1.3 to 8.6)	1.3 (0.3 to 3.1)	2.4 (1.1 to 5.1)	1.8 (1.3 to 10)	4.1 (2.4 to 7.8)

**Mortality on ICU/HDU n (%)**	0	3 (8%)	1 (4%)	4 (8%)	0	12 (16%)

Overall, 20 patients (9.1%) died during admission to HDU/ICU. Among patients with severe sepsis, 12 (16%) died compared with 4 (8%) of patients with severe non-infective SIRS. Patients with sepsis on admission to ICU had a longer median length of stay than patients with non-infective SIRS; 4.1 vs 2.4 days (*P *= 0.01).

The microbiological and infection characteristics of the sepsis patients are summarised in Table 2. The great majority of sepsis patients had a focus of infection either in the respiratory tract or abdomen. Infection was microbiologically proven in 33/87 patients with sepsis (38%) and diagnosed on radiological or clinical grounds in the remainder. Of 75 patients with non-infective SIRS, 29 were post-surgical (maxillofacial, urological), 11 cardiac (surgery or out-of-hospital cardiac arrest), 9 trauma and the remainder had a mixture of medical pathologies, (drug overdose, gastro-intestinal haemorrhage, diabetic ketoacidosis, acute asthma, seizures, pulmonary embolism, dehydration).

**Table 2:** Microbiological and infection characteristics of the sepsis patients

Variable	Non-severe sepsis *N *= 11	Severe sepsis *N *= 76	Total *N *= 87
**Assessment of infection**			

**Microbiologically proven**	**4 (36%)**	**29 (38%)**	**33 (38%)**
Gram-positive bacterial	2	4	6
Gram-negative bacterial	2	15	17
Polymicrobial	0	1	1
Viral	0	9^a^	9

**Radiological diagnosis**	**0 (0%)**	**8 (11%)**	**8 (9%)**

**Clinical diagnosis**	**7 (64%)**	**39 (51%)**	**46 (53%)**
Bacteraemic	2 (18%)	5 (7%)	7 (8%)

**Focus of infection**			
Respiratory tract	1 (9%)	32 (42%)	33 (38%)
Abdomen	8 (73%)	30 (39%)	38 (44%)
Urinary tract	0 (0%)	5 (7%)	5 (6%)
Skin and soft tissue	2 (18%)	5 (7%)	7 (8%)
Other	0 (0%)	4 (5%)	4 (5%)

^a^Seven patients had proven influenza A and may have had bacterial superinfection but this was not proven microbiologically, one had parainfluenza and another Respiratory Syncytial Virus infection.

### Biomarker levels

C-reactive protein was measured for clinical purposes in 143 of the study subjects and this information was available to the clinicians categorising subjects for the study. Consequently, the performance of CRP as a biomarker could not be assessed. Of note though, while levels of CRP measured in the study samples were markedly higher in patients with sepsis than in patients with non-infective SIRS (146 (105 to 203) vs 9.5 (3.4 to 19.9) (*P *< 0.001)), there was no difference in CRP depending on severity of illness (data not shown).

**Figure 1:**
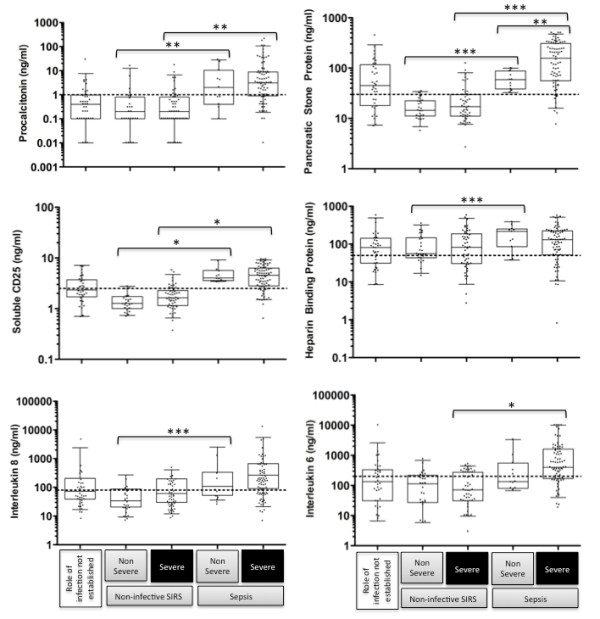
**Biomarker levels in patients with SIRS at the time of admission to intensive/high dependency care**. Boxes show medians and IQRs, whiskers show the 5^th ^and 95^th ^percentiles. Dotted horizontal lines show cut-offs defined by ROC analysis for distinguishing sepsis from non-infective SIRS. Brackets show statistically significant differences (independent samples T-test) as follows; * *P *< 0.001, **P < 0.01, *** *P *< 0.05).

Plasma levels of the different biomarkers measured in the study are shown in Figure 1. Levels of PCT (3.1 (0.8 to 3.9) vs 0.2 (0.1 to 0.8) (*P *< 0.001)), sCD25, (4.5 (3.0 to 6.1) vs 1.5 (1.1 to 2.1) (*P *< 0.001)), PSP (116 (50 to 216) vs 16.5 (11.1 to 27.9) (*P *< 0.001)) and IL6 (373 (150 to 1435) vs 83.5 (31 to 261) (*P *< 0.001)) were higher in patients with sepsis than in patients with non-infective SIRS. PSP was the only marker for which levels were significantly higher in patients with severe sepsis than patients with non-severe sepsis (157 (56 to 310) vs 59 (39 to 88) (*P *= 0.01)). Levels of HBP were similar in all patient groups. Among the 36 patients who could not be classified as having sepsis or non-infective SIRS, there were no significant differences between those with severe and non-severe disease. The other cytokines tested for were only detected in a minority of patients as follows: IL1β, 84 (36.4%), GMCSF, 26 (11.9%) and TNFα 5 (2.3%). A detectable level of these markers was more common in patients with sepsis than patients with non-infective SIRS: IL1β 61% vs 15%, GMCSF 26% vs 1% and TNFα 3% vs 0%. The small proportion of sepsis patients with detectable levels of TNFα is likely to be due to the fact that samples were taken with relation to the time of admission to ICU rather than the onset of sepsis.

**Figure 2:**
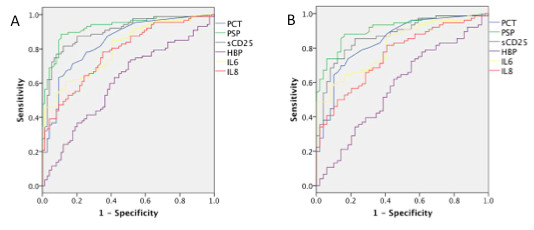
**Receiver Operating Characteristic curves for biomarkers**. **A**) 162 patients with either sepsis or non-infective SIRS. **B**) 126 patients with dysfunction of one or more organ system (SOFA score ≥ 2 and either sepsis or non-infective SIRS. Data for GMCSF and TNFα are not shown as only a minority of patients had detectable levels of these markers.

These differences were reflected in the ROC analysis shown in Figure 2. For each marker, co-ordinates of the ROC curve were used to select optimal cut-offs for distinguishing sepsis from non-infective SIRS. These cut-offs are shown on Figure 1 and the performance of each marker is set out in Table 3. Although AUCs for PSP and sCD25 were higher than for PCT in differentiating sepsis from non-infective SIRS these differences were not statistically significant

**Table 3:** Diagnostic performance of biomarkers.

Marker	AUC (95% CI)	Cut off	Sensitivity	Specificity	PPV	NPV
**PCT**	0.84 (0.78 to 0.90)	1.0 ng/ml	71%	82%	83%	71%
**PSP**	0.93 (0.89 to 0.97)	30 ng/ml	90%	83%	86%	87%
**sCD25**	0.90 (0.85 to 0.95)	2.5 ng/ml	83%	83%	85%	81%
**HBP**	0.60 (0.52 to 0.69)	50 ng/ml	78%	36%	59%	59%
**IL6**	0.81 (0.74 to 0.87)	200 pg/ml	68%	68%	71%	65%
**IL8**	0.78 (0.71 to 0.85)	80 pg/ml	78%	63%	71%	71%
**IL1β**	0.76 (0.69 to 0.84)	1.0 pg/ml	61%	88%	86%	69%

Biomarker performance is shown for 162 patients in distinguishing sepsis (*n *= 87) from SIRS without an infective aetiology (*n *= 75) Data for GMCSF and TNFα are not shown as only a minority of patients had detectable levels of these markers.

### Relationship between biomarkers and severity of illness

We set out to assess the performance of each marker in terms of reflecting illness severity first by analysing those 126 patients who had severe sepsis or non-infective SIRS (severe being defined by a SOFA score of 2 or more for any organ system). Of these 126 patients, 76 had severe sepsis and 50 severe non-infective SIRS. By ROC analysis PSP, sCD25 and PCT all performed well with AUCs over 0.8 (Figure 2). The performance of each marker is set out in Table 4. The AUCs for PSP and sCD25 were again higher than for PCT but these differences were not statistically significant.

**Table 4:** Diagnostic performance of biomarkers in distinguishing sepsis from non-infective SIRS in patients with organ dysfunction.

Marker	AUC (95% CI)	Cut off	Sensitivity	Specificity	PPV	NPV
**PCT**	0.84 (0.77 to 0.91)	1.0 ng/ml	74%	81%	86%	67%
**PSP**	0.91 (0.86 to 0.96)	30 ng/ml	88%	78%	86%	81%
**sCD25**	0.87 (0.81 to 0.93)	2.5 ng/ml	80%	78%	85%	72%
**HBP**	0.58 (0.48 to 0.68)	50 ng/ml	78%	38%	66%	53%
**IL6**	0.82 (0.74 to 0.89)	200 pg/ml	71%	66%	76%	60%
**IL8**	0.76 (0.68 to 0.84)	80 pg/ml	82%	58%	75%	67%
**IL1β**	0.77 (0.69 to 0.85)	1.0 pg/ml	65%	88%	89%	62%

Biomarker performance is shown for 76 patients with severe sepsis and 50 patients with non-infective SIRS and organ dysfunction. Data for GMCSF and TNFα are not shown as only a minority of patients had detectable levels of these markers.

**Figure 3:**
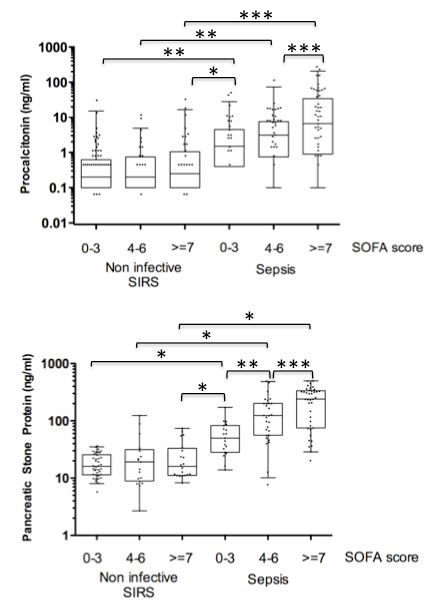
**Relationship between biomarker levels and severity of illness**. Boxes show medians and IQRs, whiskers show 5^th ^and 95^th ^percentiles. Brackets show statistically significant differences in pairwise group comparisons (independent samples T-test) as follows; * *P *< 0.001, **P < 0.01, *** *P *< 0.05).

To look more closely at the ability of biomarkers to reflect severity of illness, patients with non-infective SIRS and sepsis were grouped according to SOFA score with severity defined as mild (SOFA 0 to 3), moderate (SOFA 4 to 6) and severe (SOFA > = 7). For both PCT and PSP there was a clear relationship between plasma level and severity of sepsis but not severity of non-infective SIRS (Figure 3). Focusing on patients with SOFA score > = 7, the AUC for PSP in discrimination of sepsis from non-infective SIRS was higher at 0.955 (0.909 to 1) vs 0.837 (0.732 to 0.941). However, this difference was not statistically significant.

### Regression analysis and evaluation of combined performance of biomarkers

In univariate regression analysis, for both PSP (cut-off 30 ng/ml) and sCD25 (cut-off 2.5 ng/ml), elevation was strongly associated with sepsis compared with non-infective SIRS and severe sepsis compared with non-severe SIRS of non-infective aetiology (Table 5). For both markers, the odds ratios for sepsis were higher than for PCT but not-statistically significantly so as the 95% confidence intervals overlap. Elevation of HBP above the cut-off of 50 ng/ml was not statistically associated with either sepsis or severe sepsis.

**Table 5:** Biomarker levels according study group.

	Cut off	SIRS *N *= 75	Sepsis *N *= 87	OR	***P***	Severe SIRS *N *= 50	Severe sepsis *N *= 76	OR	***P***
**PCT**	1.0 ng/ml	13/74*	62/87	11.6 (5.5 to 24.8)	< 0.0001	9/49*	56/76	12.4 (5.1 to 30.2)	< 0.0001
**PSP**	30 ng/ml	13/75	78/87	41.3 (16.6 to 103.0)	< 0.0001	11/50	67/76	26.4 (10.1 to 69.3)	< 0.0001
**sCD25**	2.5 ng/ml	13/75	72/87	22.9 (10.1 to 51.8)	< 0.0001	11/50	61/76	14.4 (6.0 to 34.7)	< 0.0001
**HBP**	50 ng/ml	48/75	68/87	2.0 (1.0 to 4.0)	0.06	31/50	59/76	2.1 (1.0 to 4.7)	0.07
**IL6**	200 ng/ml	24/75	59/87	4.5 (2.3 to 8.7)	< 0.0001	17/50	54/76	4.8 (2.2 to 10.3)	< 0.0001
**IL8**	80 ng/ml	28/75	68/87	6.0 (3.0 to 12.0)	< 0.0001	21/50	62/76	6.1 (2.7 to 13.7)	< 0.0001
**IL1b**	1.0 ng/ml	9/75	57/87	13.9 (6.1 to 31.8)	< 0.0001	6/50	49/76	13.3 (5.5 to 35.3)	< 0.0001

Univariate odds ratios (95% confidence intervals) are given for sepsis or severe sepsis using cut-offs derived by ROC analysis. * One patient with severe SIRS did not have PCT measured.

Calculation of correlation coefficients for the different variables revealed a marked degree of correlation between the markers (see Additional file 1). Correlation coefficients for PSP, sCD25 and IL6 with PCT were between 0.55 and 0.59. HBP showed poor correlation with other markers. Markers for which the ROC was > 0.8 were selected to include in the logistic regression analysis (PSP, sCD25 and IL6 with PCT). In a forward, stepwise, logistic regression analysis, the addition of sCD25 improved the performance of both PCT and PSP, improving the AUC of these markers alone for differentiating severe sepsis from severe non-infective SIRS (Table 4) from 0.84 and 0.91 to 0.89 (0.83 to 0.95) and 0.94 (0.90 to 0.98), respectively. Among patients with severe sepsis, 56 had levels of both PSP and sCD25 above the cut-off while only 4 had levels below the cut-off for both markers. In 16 patients, the markers were discordant. Conversely, in patients with severe non-infective SIRS only 2 patients had elevation of both markers while 31 had levels of both below the cut-off. In 17 patients, the markers were discordant. The negative predictive value of low PSP and low sCD25 was thus 89% and the positive predictive value of high PSP and high sCD25 was 97%, with 33/126 (26.2%) having discordant results. No improvement in the sensitivity or specificity was observed with other combinations of markers.

## Discussion

The main findings of this study, performed in patients at the time of admission to intensive or high dependency care, are: 1) that two recently described sepsis biomarkers (PSP and sCD25) perform at least comparably with PCT in identifying patients with sepsis; 2) PSP shares with PCT the property of reflecting sepsis severity; 3) HBP, despite promise in other settings, does not appear to perform well in this population; 4) a combinatorial approach to biomarker use might offer better discrimination in sepsis than use of a single biomarker alone.

Our study has several important strengths. Unlike the majority of sepsis biomarker studies which enrol very specific patient groups, we have tested these markers in unselected patients with the diverse range of pathologies, infective and otherwise, which present to high dependency and intensive care units. By using well established and objective outcome categories and incorporating all available clinical information we have been able to define groups of patients with either sepsis or non-infective SIRS. This has allowed us to assess the biomarkers without including clinically unrealistic control patients without suspected infection or sepsis.

Previous studies of PSP have reported elevated levels in patients following non-pancreatic trauma which reflect severity in trauma patients who develop infection [10]. In patients with ventilator-associated pneumonia, levels of PSP at the time of diagnosis correlate with severity of illness and survival [12]. In keeping with these observations we find that while most patients at the time of admission to intensive care have elevated PSP levels, this does reflect the presence of sepsis and severity of illness to a degree which is comparable to PCT. We excluded from this analysis four patients with acute pancreatitis not because of the possible effect of this on PSP level but because it was not possible in these patients to be certain whether infection was present. Notably, all four patients had elevated levels of PSP. We did not exclude from the analysis patients with renal impairment or gastrointestinal pathologies.

Levels of sCD25 in the blood appear to reflect the level of CD25 expression on activated T cells which has been suggested as a marker of activation-induced regulatory T cell response [14]. Whereas levels of acute phase reactants, such as PCT and PSP, reflect the magnitude of an inflammatory response, expression of CD25 may reflect the development of a compensatory anti-inflammatory state and thus provide additional information about an individual's response to sepsis at a point in time [13]. Our findings are in keeping with those of Saito *et al. *who found higher levels of sCD25 in 20 sepsis patients than 16 patients with non-infective SIRS [14]. More recently, Lvovschi *et al. *performed multiplex cytokine analysis on 126 patients presenting to the emergency department with non-infective SIRS or sepsis [21]. Although they were unable to demonstrate profiles characteristic of sepsis, sCD25 was the only marker independently associated with severe sepsis in multivariate analysis. Interestingly, sCD25 levels were generally lower among the patients in that study (median value < 1 ng/ml) and lower among patients with severe sepsis than in patients with mild disease. These differences probably reflect sampling time relative to the onset of sepsis and emphasise the importance of not extrapolating data from sepsis biomarker studies performed in one setting to another.

The poor performance of HBP in differentiating sepsis from non-infective SIRS in this study suggests that the value of this marker should be further explored in the emergency department rather than in the intensive care setting. Linder *et al. *measured HBP in febrile adults presenting to the emergency department and showed that levels ≥ 15 ng/ml were a better indicator of severe sepsis or septic shock than procalcitonin [15]. Subsequent studies by the same group have demonstrated elevated levels of HBP in cerebrospinal fluid of patients with bacterial meningitis and the urine of children with urinary tract infections [22,23]. Our findings contrast with three previous studies of HBP conducted in ICU. Berkestedt *et al. *reported elevated HBP levels correlating with severity in 33 patients with severe sepsis [24], while Chew *et al. *found elevated levels of HBP in 53 patients with shock on the ICU irrespective of infectious aetiology and no correlation with severity and outcome [25]. More recently, Linder *et al. *reported higher HBP levels in patients with sepsis than with non-septic critical illness on ICU and a correlation with mortality. However, 50% of control patients had elevated HBP levels. It is possible that the lack of a relationship between HBP level and infection or severity of illness in our study relates to the unselected nature of the patients we recruited.

Other studies have reported the use of combinations of sepsis biomarkers to increase diagnostic accuracy driven in part by the availability of multiplex platforms allowing panels of markers to be assessed simultaneously. The high degree of correlation which we and others have observed between inflammatory markers represents the main challenge to a combinatorial approach [21]. The increase in accuracy we observe from adding sCD25 to PSP or PCT raises the possibility of increased value that can be gained from choosing markers of different arms of the inflammatory response (neutrophils, acute phase proteins, cytokines, T cell activation/regulatory markers).

Our study has some important limitations. To perform ROC analysis and calculate predictive values for each marker, we have had to classify patients as having sepsis or non-infective SIRS, but as a result of the study being observational and having unselected entry criteria, 18% of patients with SIRS could not be robustly characterised as having either sepsis or a non-infective aetiology. While this is the patient group in which a novel marker might be most valuable, our study was not designed to address this. The studies needed to establish whether PSP or sCD25 could improve diagnosis in this most challenging patient group would require protocolised investigation of patients and multicentre recruitment. They would thus be expensive and time consuming. Our findings indicate that PSP and sCD25 may be good candidates to take forward into such studies.

An additional limitation is that we have assessed the markers' performance in identifying sepsis and severe sepsis only at the time of sampling. Thus, we cannot draw conclusions about the predictive value of the markers for the development of sepsis at a later time or assess the impact of serial measurements. Furthermore, the average SOFA score of patients in our study is relatively low considering this is an ICU-based sepsis study as is the proportion of sepsis patients where a firm microbiological diagnosis was made. Both of these criticisms are inherent in the study's design which prioritised recruitment of unselected subjects at the time of admission over long-term follow-up of highly selected patients. The advantage of this approach is that it has allowed us to perform a challenging, real-life assessment of these markers which should now inform the design of the larger studies, with more detailed follow-up, which are required to determine which markers could predict the development of sepsis or the progression of severity in sepsis for patients in ICU or HDU.

## Conclusions

Candidate sepsis biomarkers are often first described in studies focussing on selected patient groups and fail to translate into routine clinical practice. We have performed an assessment of three promising markers among unselected patients with a systemic inflammatory response and in a clinical setting where sepsis biomarkers are commonly used, at the time of a patient's admission to intensive or high dependency care. Two markers (PSP and sCD25) performed well in this setting, while the third (HBP) did not. Our findings support the further assessment of PSP and sCD25 to inform clinical decision making in patients with suspected sepsis in the high-dependency care setting.

## Key messages

• Candidate sepsis biomarkers that show promise in highly selected patient groups should be validated in studies which recruit unselected patients more typical of clinical practice. This approach represents an important stepping-stone between the first description of a marker and its validation in the time-consuming and expensive intervention studies, which are required for introduction to clinical practice.

• Two of the markers studied here, PSP and sCD25, perform well in differentiating sepsis from non-infective illnesses and warrant further study in critical care patients. The third, HBP, does not appear to perform well in this setting.

• The performance of sepsis biomarkers may vary between clinical settings, such as the emergency room and the intensive care unit.

## Abbreviations

AUC: Area Under the Curve; CRP: C-Reactive Protein; GMCSF: Granulocyte Macrophage Colony Stimulating Factor; HBP: Heparin Binding Protein; HDU: High Dependency Unit; ICU: Intensive Care Unit; IQR: Interquartile Range; IL: Interleukin; PCT: Procalcitonin; PSP: Pancreatic Stone Protein; ROC: Receiver Operating Characteristic; sCD25: Soluble CD25; SIRS: Systemic Inflammatory Response Syndrome; SOFA: Sequential Organ Failure Assessment score; TNFα: Tumour Necrosis Factor alpha

## Competing interests

This work was funded by Abbott GmbH & Co. KG, Wiesbaden, Germany. MB, IC and SB are employees of Abbott GmbH & Co. KG Wiesbaden, Germany and hold Abbott Laboratories shares. FL is a shareholder of Lascco SA, Geneva, Switzerland, which has rights to license PSP worldwide. RG is an inventor of the PSP assay for the determination of sepsis for which the University of Zurich is the patent holder. The University of Zurich is owner of the patent for this invention and RG receives a percentage of the proceeds from licensing agreements between the University of Zurich and sub licensees. Abbott Laboratories and the University of Sussex are applying for a patent that covers the use of PSP in sepsis. Successful intellectual property applications are financially rewarded within Abbott. All other authors declare no conflicts of interest.

## Authors' contributions

ML, SB, IC, SD and JC designed the study. ML and SD categorised the subjects. MG recruited the subjects and, with RR, compiled the clinical data. AT was responsible for measuring sCD25 levels. FL and RG were responsible for measurement of PSP levels. ML, MB and AT analysed the data. ML wrote the manuscript and is custodian of the data. All authors have read and approve the final manuscript.

## Supplementary Material

Additional file 1 **Correlation between biomarkers**. A table showing correlations for all the biomarkers and inflammatory mediators measured. For each, correlation coefficients are shown. Correlation coefficients greater than 0.5 are highlighted in red. Click here for file
