# Supplementary material for: Sepsis biomarkers in unselected patients on admission to intensive or high-dependency care
Source: Crit Care. 2013 Mar 26;17(2):R60. doi: 10.1186/cc12588 (PMC3672658; doi:10.1186/cc12588)
Supplement: Additional file 1 — Correlation between biomarkers. A table showing correlations for all the biomarkers and inflammatory mediators measured. For each, correlation coefficients are shown. Correlation coefficients greater than 0.5 are highlighted in red. [file cc12588-S1.DOCX]

**Additional Table. Correlation between biomarkers.** Correlation coefficients greater than 0.5 are highlighted in red.

|  |  | **PSP** | **sCD25** | **PCT** | **HBP** | **IL6** | **IL8** | **IL1β** | **GMCSF** |
| --- | --- | --- | --- | --- | --- | --- | --- | --- | --- |
| **sCD25** | **rho** | **0.584** |  |  |  |  |  |  |  |
|  | p | <0.001 |  |  |  |  |  |  |  |
| **PCT** | **rho** | **0.591** | **0.546** |  |  |  |  |  |  |
|  | p | <0.001 | <0.001 |  |  |  |  |  |  |
| **HBP** | **rho** | **0.092** | **0.169** | **0.211** |  |  |  |  |  |
|  | p | 0.18 | 0.01 | <0.001 |  |  |  |  |  |
| **IL6** | **rho** | **0.481** | **0.347** | **0.570** | **0.082** |  |  |  |  |
|  | p | <0.001 | <0.001 | <0.001 | 0.23 |  |  |  |  |
| **IL8** | **rho** | **0.535** | **0.383** | **0.456** | **0.123** | **0.614** |  |  |  |
|  | p | <0.001 | <0.001 | <0.001 | 0.07 | <0.001 |  |  |  |
| **IL1β** | **rho** | **0.403** | **0.341** | **0.431** | **0.109** | **0.731** | **0.534** |  |  |
|  | p | <0.001 | <0.001 | <0.001 | 0.11 | <0.001 | <0.001 |  |  |
| **GMCSF** | **rho** | **0.350** | **0.291** | **0.417** | **0.027** | **0.545** | **0.467** | **0.589** |  |
|  | p | <0.001 | <0.001 | <0.001 | 0.69 | <0.001 | <0.001 | <0.001 |  |
| **TNFα** | **rho** | **0.079** | **0.096** | **0.042** | **0.054** | **0.096** | **0.058** | **0.213** | **0.041** |
|  | p | 0.24 | 0.16 | 0.54 | 0.43 | 0.16 | 0.39 | <0.001 | 0.55 |
